# A Bispecific Antibody-Based Approach for Targeting Mesothelin in Triple Negative Breast Cancer

**DOI:** 10.3389/fimmu.2019.01593

**Published:** 2019-07-10

**Authors:** Joanie Del Bano, Rémy Florès-Florès, Emmanuelle Josselin, Armelle Goubard, Laetitia Ganier, Rémy Castellano, Patrick Chames, Daniel Baty, Brigitte Kerfelec

**Affiliations:** ^1^Aix Marseille Univ, CNRS, INSERM, Institut Paoli-Calmette, CRCM, Marseille, France; ^2^Aix Marseille Univ, CNRS, Institut de Biologie du Développement de Marseille, UMR7288, Marseille, France

**Keywords:** bispecific antibody (bsAb), natural killer cells, immunotherapy, triple negative breast cancer (TNBC), mesothelin (MSLN), multicellular tumor spheroid model (MCTS)

## Abstract

Triple negative breast cancers (TNBC) remain a major medical challenge due to poor prognosis and limited treatment options. Mesothelin is a glycosyl-phosphatidyl inositol-linked membrane protein with restricted normal expression and high level expression in a large proportion of TNBC, thus qualifying as an attractive target. Its overexpression in breast tumors has been recently correlated with a decreased disease-free survival and an increase of distant metastases. The objective of the study was to investigate the relevance of a bispecific antibody-based immunotherapy approach through mesothelin targeting and CD16 engagement using a Fab-like bispecific format (MesobsFab). Using two TNBC cell lines with different level of surface mesothelin and epithelial/mesenchymal phenotypes, we showed that, *in vitro*, MesobsFab promotes the recruitment and penetration of NK cells into tumor spheroids, induces potent dose-dependent cell-mediated cytotoxicity of mesothelin-positive tumor cells, cytokine secretion, and decreases cell invasiveness. MesobsFab was able to induce cytotoxicity in resting human peripheral blood mononuclear cells (PBMC), mainly through its NK cells-mediated antibody dependent cell cytotoxicity (ADCC) activity. *In vivo*, the anti-tumor effect of MesobsFab depends upon a threshold of MSLN density on target cells. Collectively our data support mesothelin as a relevant therapeutic target for the subset of TNBC that overexpresses mesothelin characterized by a low overall and disease-free survival as well as the potential of MesobsFab as antibody-based immunotherapeutics.

## Introduction

Over the past 15 years, the enrichment of the diagnosis and therapeutic arsenal against breast cancers has undoubtedly contributed to improving their prognosis. However, despite these advances, recurrences still occur and metastatic breast cancer remains in most cases an incurable disease. Moreover, therapeutic failures are still frequent either because of intrinsic or acquired resistance to treatment, especially in the case of so-called triple-negative breast cancers (TNBC). Indeed TNBC which represent 10–20% of invasive breast cancers account for the majority of deaths in breast cancer patients due to a higher recurrence rate and a worse overall survival rate than other subtypes of breast cancer (1). Apart from their aggressiveness and their ability to rapidly metastasize, these cancers are characterized by a strong heterogeneity and the lack of markers such as HER2, estrogen and progesterone receptors, and a variable response to treatment. Treatment options still remain limited with chemotherapy being the standard of care treatment. Therefore, the development of new strategies and/or new therapeutic molecules for these dark prognosis cancers is a major clinical challenge.

Mesothelin (MSLN), a 40 kDa glycosyl-phosphatidyl inositol-linked (GPI) membrane glycoprotein, has been attracting growing interest in the field of cancer therapy. In healthy tissues, MSLN is only expressed on mesothelial cells lining the peritoneum, pericardium, and pleura (2). The most common form of MSLN is membrane-bound but a soluble form devoid of GPI anchor has also been described (3, 4). Apart from its presumed role in cell-cell adhesion, its biological function is still not fully understood and its knockout in mice has no detectable phenotype (5). MSLN is overexpressed in several human cancers, notably those characterized by aggressive phenotypes and poor prognosis such as mesotheliomas, pancreatobiliriary, ovarian, and lung carcinoma (6). High amount of soluble MSLN has been also reported in the serum of patients with ovarian cancer. In a tumor context, MSLN plays an important role in survival, proliferation, and migration/invasion of cancer cells as well as in drug resistance, potentially through the Wnt/NF-κB/PI3K/Akt signaling pathways (7) and/or by facilitating the anchorage-independent growth (8). An association between MSLN expression, tumor invasion and matrix metalloproteinases activation has been demonstrated *in vitro, in vivo*, and in patients with malignant pleural mesothelioma (9). Its strong interaction with MUC16 (CA125), a large membrane-spanning glycoprotein, contributes to peritoneal spread in ovarian cancer by promoting heterotypic cell adhesion (10) and increases the motility and invasiveness of pancreatic cancer cells (11). In mouse models of pancreatic cancer, MSLN overexpression promotes metastasis and proliferation of tumor cells. The causes of MSLN overexpression in cancer are not clear but could be related to the hypomethylation of MSLN gene (12) or the deregulation of the Wnt signaling pathways (13).

Confirming the interest of MSLN as therapeutic target, a growing number of clinical trials are currently ongoing at different stages for evaluating diverse MSLN targeted strategies such as vaccines, antibody drug conjugate, monoclonal antibodies (Morab-009), Meso CART-cells (6, 14, 15), or combinations (16). Overexpression of MSLN was recently demonstrated in TNBC. Depending on the scoring system, the percentage of MSLN positive TNBC among TNBC varies from 36 to 63% (17, 18) and although controversial (19, 20), a correlation with lymph nodes infiltration and poor overall survival has been described in MSLN-positive TNBC (18).

In this study, we exploited a previously described single domain-based bispecific antibody Fab-like format (21, 22) to generate a bispecific antibody targeting MSLN and FcγRIII (CD16), one of the strongest activating receptor notably expressed by NK cells. Its antitumor efficacy was evaluated *in vitro* on 2D (monolayer) and 3D (spheroid) TNBC culture models and *in vivo* in two TNBC xenograft mice models.

## Results

### Design and Characterization of Anti-MSLN x CD16 Bispecific Antibody

Based on our proprietary Fab-like bispecific format (bsFab) and previously characterized anti-MSLN [clone A1, (23)] and anti-CD16 [clone C21, (24)] nanobodies, we designed a bsFab (MesobsFab) targeting mesothelin positive tumor cells and CD16 positive immune cells (Figure S1A). Binding properties of MesobsFab were investigated by flow cytometry on HCC1806 cell line and on human CD16-transfected Jurkat cells (Figure S1). Consistent with our previous data (21, 22), MesobsFab exhibited a high apparent affinity for CD16 with a low K_Dapp_ value (1.8 ± 0.8 nM), compared to that of conventional human IgG Fc fragment (>100 nM) (Table S1). The K_Dapp_ values on HCC1806 cells (4.7 ± 0.9 nM) were in good agreement with previous data obtained with the anti-MSLN sdAb A1 on HELA cells (23), indicating that the specific binding activity of this sdAb was retained in the bispecific format. MesobsFab specificity was also confirmed by competition assays (Figure S1).

### MesobsFab Decreases the Invasive Properties of TNBC Cell Lines *in vitro*

**Figure 1:**
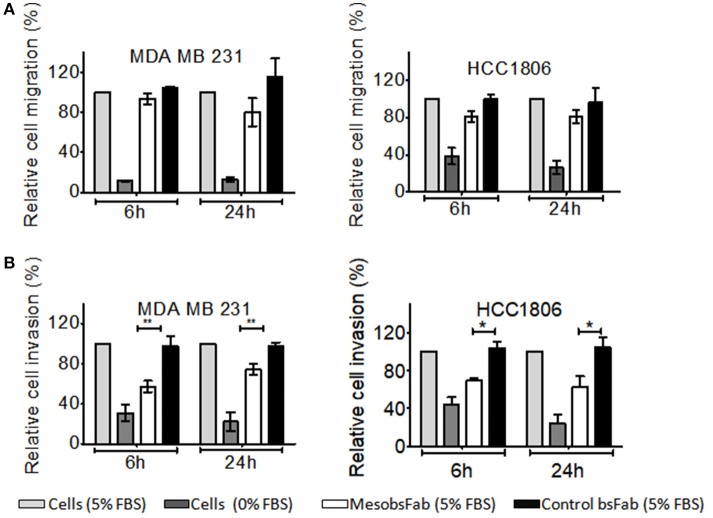
MesobsFab binding to mesothelin reduces HCC1806 invasiveness. Effect of MesobsFab or control bsFab (50 nM) on the migration and invasion of MDA MB 231 and HCC1806 cells. **(A)** CFSE-stained tumor cells were allowed to migrate toward culture medium supplemented with 5% FBS for 6 or 24 h at 37°C. The fluorescence signals measured in the Fluoroblok bottom chambers correspond to migrating cells. A control of low migration was performed by omitting the FBS in the culture medium. **(B)** CFSE-stained MDA MB 231 and HCC1806 cells were seeded onto a matrigel coated fluoroblok inserts and allowed to migrate in response to serum gradient. 100% inhibition corresponds to the absence of CFSE-stained tumor cells in the bottom chamber. In all panels, data represent the mean ± SEM from 3 independent experiments. Statistical significance was determined by two-tailed Student's *t*-test: ^*^P < 0.05, ^**^P < 0.001, MesobsFab vs. control bsFab.

MSLN overexpression has been reported to be associated with a strongly invasive and aggressive tumor phenotype in TNBC. Based on the hypothesis that the binding of MesoFab to MSLN might interfere with this property, we investigated the potential effects of MesobsFab on the *in vitro* migration/invasion properties of MDA MB 231 and HCC1807 cells. MesobsFab displayed a reproducible tendency to slightly decrease the migration of HCC1806 and MDA MB 231 cells without reaching significance (Figure 1A). By contrast, a significant decrease of both MDA MB 231 and HCC1806 invasiveness was observed in the presence of MesobsFab (Figure 1B).

### Formation of Homotypic Multicellular Tumor Spheroids Derived From TNBC Cells

**Figure 2:**
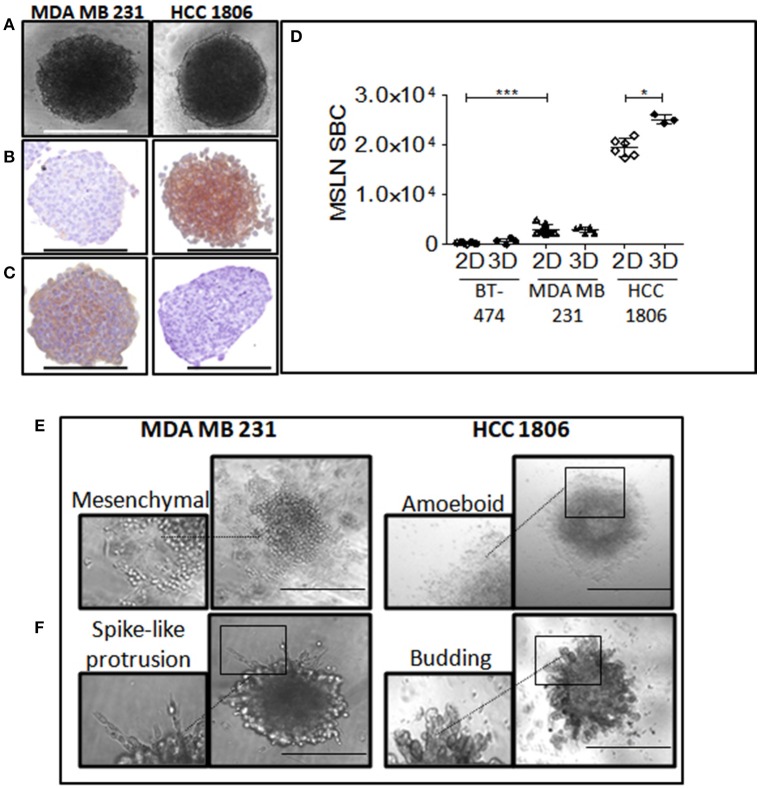
Characterization and phenotypic properties of TNBC spheroids. **(A)** Representative bright field images of MDA MB 231 and HCC1806 spheroids. **(B,C)** Epithelial/mesenchymal phenotypes of MDA MB 231 and HCC1806 spheroids. Representative images of E-cadherin **(B)** and vimentin **(C)** staining of histological sections of 7 day- spheroids. Scale bar, 500 μm. **(D)** Specific MSLN binding capacities of 2D and 3D MDA MB 231, HCC1806, and BT474 cell cultures. Values with errors bars represent mean ± SEM (*n* = 3). Data were analyzed by two-tailed Student's *t*-test. ^*^P < 0.05, HCC1806 2D vs. 3D models; ^***^P < 0.001, 2D MDA MB 231 vs. BT474. **(E,F)** Migration/invasion patterns of MDA MB 231 and HCC1806 spheroids. Representative images of migration **(E)** and invasion **(F)** pattern of 4 days spheroids were transferred on 1% gelatin or embedded in 50% matrigel, respectively, and cultured for 2 days at 37°C before imaging. Images were acquired with an EVOS FL Auto microscope. Scale bar, 500 μm.

Homotypic spheroids were generated from the two TNBC cell lines using the static liquid overlay method (3, 25). Growth of spheroids and changes in morphology were monitored by phase contrast light microscopy. TNBC cells formed cell clusters within 24 h after seeding and reached a characteristic 3D organization after 2–4 days as shown by the formation of more or less compact and round-shaped spheroids and the disappearance of cells in suspension in the growth medium. The mean radius of 4-days spheroids (CV > 10%) was similar for MDA MB 231 and HCC1806 spheroids (222.9 ± 16.8 vs. 224.1 ± 17.9 μm, respectively). HCC1806 spheroids displayed a rather rounded and compact morphology while MDA MB 231 spheroids were less regular and less compact likely due to weaker cell-cell contacts (Figure 2A). As described in the literature, the spheroid periphery consisted of viable cells while necrotic cells were located in the core as evidenced by Hoechst 3342 and Propidium Iodide (PI) staining (Figure S2A). Evolution of cell death during spheroid growth was monitored by PI staining at different time points, revealing a discrete area of necrosis already at day 4 which increased over time (Figures S2B,C). The necrosis process was more pronounced in the HCC1806-spheroids than in the MDA MB 231-spheroids and was accompanied by a visible cellular migration phenomenon. Epithelial/mesenchymal phenotypes of TNBC spheroids were investigated by immunochemistry on 7-day spheroids through the expression of epithelial (E-cadherin) or mesenchymal (Vimentin) markers. MDA MB 231 spheroids presented a high vimentin staining (Figure 2B) and a low E-cadherin expression (Figure 2C) characterizing a mesenchymal-like phenotype while HCC1806 spheroids displayed a strong E-cadherin staining and a lack of vimentin expression suggesting an epithelial phenotype.

The specific antibody binding capacity (SABC) reflecting the antigen density of MDA MB 231 cells was significantly lower than that of HCC1806 but still significantly higher than that of MSLN-negative cells BT-474 (Figure 2D). A slight but significant (*p* < 0.05) increase of MSLN binding capacity was observed on HCC1806 spheroids compared to 2D monolayers.

Analysis of migration/invasion patterns demonstrated that MDA MB 231 cells disseminated outside the spheroids mainly as cell clusters and displayed heterogeneous rounded and elongated morphologies (Figure 2E). In the presence of matrigel, MDA MB 231 spheroids formed invasive spike-like protrusions (Figure 2F), typical of mesenchymal-like phenotype. By contrast, the migration/invasion pattern of HCC1806 cells in 3D resembled amoeboid migration and bud-like invasion phenotype.

### NK Cells Recruitment and Infiltration Into TNBC Spheroid

**Figure 3:**
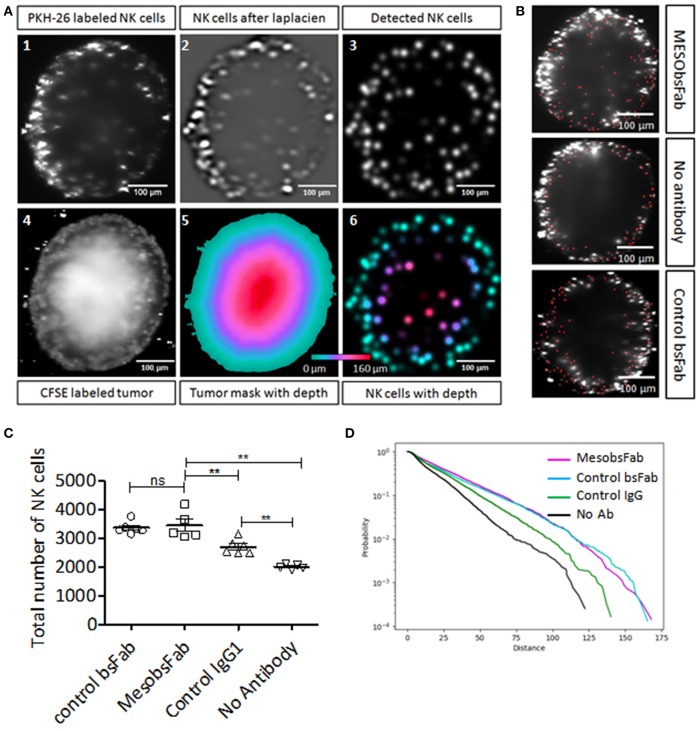
Impact of MesobsFab on NK cells infiltration into TNBC spheroids. **(A)** Cross-sections illustrating the 3D distance analysis method using ImageJ software. Multi-views images of spheroid were fused at 488 nm for visualizing CFSE-labeled tumor cells(A.4) and 561 nm for PKH-26-labeled NK cells (A.1). A Laplacian of Gaussian filter was applied to spatially filter the NK cells (A.2), then the NK cells were detected by measuring local maxima (A.3). A 3D mask (A.5) was created based on CFSE-labeled tumor cells (A.4) and used for measuring the distance of each NK cells from the spheroid surface (A.6) by computing the mask distance transform (A.5). **(B)** Representative images of infiltrated NK cells (red dots) into spheroid treated by MesobsFab, control bsFab, or without antibody. **(C)** Total number of NK cell detected into HCC1806 spheroid after 2 days of co-culture (E:T ratio 2.5:1) in the presence or absence of bsFab (50 nM). **(D)** Cumulative distribution curve representing the probability of NK cell infiltration based on the distance penetration. All spheroids size were homogeneous (CV < 6%) and had a radius size of 188.4 ± 10, 7 μm (*n* = 5–6/group). Data points represent the mean ± SEM and were analyzed by two-tailed Student's *t*-test. ^**^P < 0.01 MesobsFab vs. no Ab.

Human resting NK cell recruitment and infiltration mediated by MesobsFab were investigated on HCC1806 spheroids by light-sheet fluorescence microscopy. CFSE-stained HCC1806 spheroids were co-cultured with PKH-26 stained NK cells with or without MesobsFab or control bsFab. After spheroid reconstruction, qualitative and quantitative analysis were performed as depicted in Figure 3A for MesobsFab and control bsFab or IgG (Movie S1) using 3D analysis method on ImageJ software. NK cells infiltration was evaluated on 3D reconstructed images by measuring the distance of each NK cell from the spheroid surface and not from the spheroid center to overcome spheroid sphericity problems. NK cells (red dots) spontaneously infiltrated HCC1806 spheroids and most NK cells accumulated at the periphery of the tumor spheroids (Figure 3B). A significantly higher recruitment of NK cells was observed in the presence of MesobsFab, compared with control conditions without antibody (Figure 3C). Surprisingly, the control antibodies (bsFab or human IgG) also induced a significant NK cells recruitment. The control bsFab behaved similarly to MesobsFab while IgG was less effective. Using a cumulative distribution curve (Figure 3D) we showed that the probability to find NK cells deeper into the spheroid was higher in the presence of MesobsFab or control bsFab, and to lesser extent in the presence of control IgG compared to the absence of antibody.

### *In vitro* MesobsFab Mediates ADCC Against 2D and 3D TNBC Cell Culture Models

**Figure 4:**
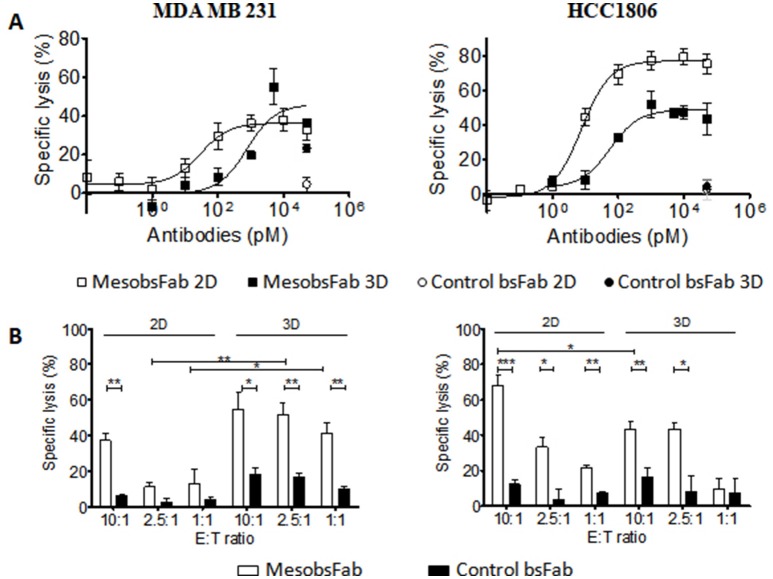
MesobsFab mediates potent *in vitro* ADCC activity against TNBC cell lines. **(A)** ADCC assays were performed on 2D and 3D cell models using serial dilutions of MesobsFab, unstimulated NK cells as effector cells and MDA MB 231 or HCC1806 as target cells (E:T ratio 10:1). Control bsFab was tested at the highest concentration (50 nM). Target cell viability was determined by CellTiter-Glo viability assay for 2D ADCC or by flow cytometry after spheroid dissociation for 3D ADCC. **(B)** Impact of the E:T ratios on the maximal lysis of MDA MB 231 and HCC 1806 monolayers and spheroids mediated by 5 nM, (EC90) MesobsFab or control bsFab. Data shown are mean ± SEM from 3 independent experiments (3 donors). Statistical analysis was performed by two-tailed Student's *t*-test. ^*^p < 0.05; ^**^p < 0.01; ^***^p < 0.001.

The capacity of MesobsFab to mediate antibody dependent cell cytotoxicity (ADCC) was evaluated on both tumor cell monolayers and spheroids. In both cases, tumor cells were co-incubated with unstimulated human NK cells for 12 h and target cell viability was measured either by CellTiter-Glo viability assay for 2D cell culture or by flow cytometry for 3D culture system. Before 3D experiments, the size homogeneity of all CFSE-stained spheroids was checked using ImageJ software. MesobsFab elicited NK cell cytotoxic in a dose-dependent manner at an effector:target (E:T) ratio of 10:1 on all culture models but differences were noticed (Figure 4A). In agreement with MSLN expression level, MesobsFab displayed a higher efficacy (maximal lysis) on HCC1806 than on MDA MB 231 monolayers (77% for HCC1806 vs. 35% for MDA MB 231) but this difference faded when cells were grown as spheroids (Lysis max: 46–49%). MesobsFab was as efficient against MDA MB 231 spheroids than against 2D monolayers, with a maximal specific lysis ranging from 35 to 46% but its potency was affected as evidenced by the large shift of the EC_50_ values on spheroids (26 vs. 919 pM). On the other hand, both the efficacy and the potency of MesobsFab decreased on HCC1806 spheroids compared to 2D monolayers, despite a higher MSLN binding capacity. Of note, a slightly higher spontaneous NK cells-mediated lysis on spheroids was observed, reaching about 30% (vs. 10–15% on monolayers). As previously described, engagement of CD16 in MesobsFab-driven ADCC was confirmed by measuring extracellular granzyme B (Figure S3A). The specificity of MesobsFab-mediated ADCC was demonstrated using either a control bsFab at the highest concentration 50 nM (Figure 4A) or MSLN-negative tumor cells (Figure S3B). Noticeable differences were observed between the 2 TNBC cell lines when the impact of E:T ratio was investigated (Figure 4B). Indeed, MesobsFab retained its cytotoxic activity at low E:T ratio on MDA MB 231 spheroids compared to control bsFab whereas its activity dropped rapidly with the E:T ratio on 2D cultures. By contrast, MesobsFab efficacy against HCC1806 was significantly higher than that of the control bsFab at E:T ratio of 10 and 2.5, regardless of the cell growth conditions. A slight increase of tumor lysis mediated by the control bsFab was observed at high E:T ratio on spheroids.

**Figure 5:**
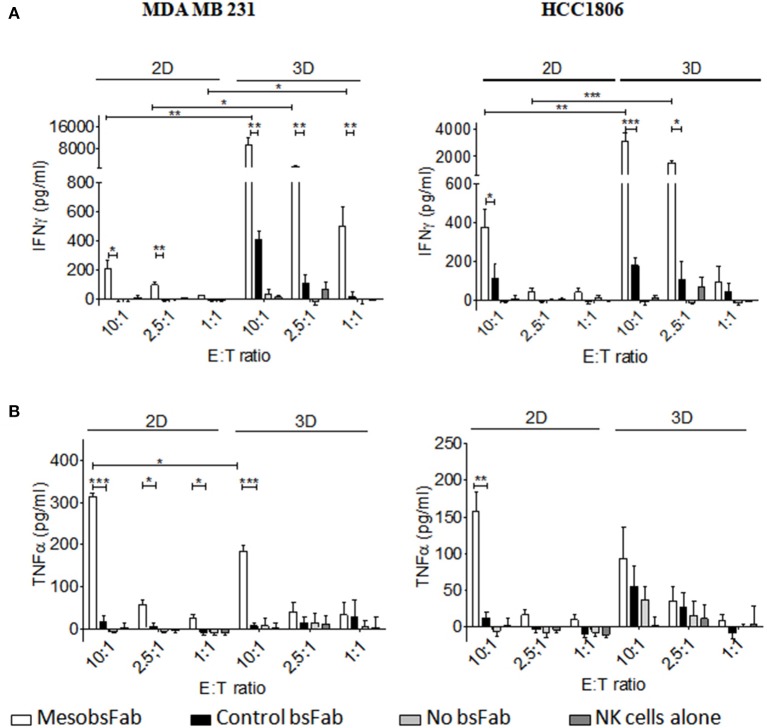
MesobsFab mediates potent IFNγ secretion. IFNγ **(A)** and TNFα **(B)** were quantified by ELISA in the culture medium harvested from ADCC assays. Experiments, performed in technical triplicate, are representative of at least three independent donors. Data represent the mean ± SEM and were analyzed by two-tailed Student's *t*-test. MesobsFab vs. control bsFab: ^*^P < 0.05, ^**^P < 0.01, ^***^P < 0.001; MesobsFab 2D vs. MesobsFab 3D ^*^P < 0.05, ^**^P < 0.01, ^***^P < 0.001.

Finally, the activation status of NK cells harvested from the above 2D and 3D ADCC assays was evaluated by measuring the secretion of inflammatory cytokines, TNFα and INFγ. The tumor cell growth conditions had a strong influence on the level of IFNγ secretion with a substantially higher MesobsFab-mediated release of IFNγ during 3D ADCC (10 to 40-fold) compared to 2D ADCC at different E:T ratio (Figure 5A). This result was observed at all E:T ratio with MDA MB 231-derived spheroids. At E:T ratio of 10, the control bsFab did lead to a small but significant IFNγ secretion in the 3D but not in the 2D model compared to NK cell alone. However, this secretion remained significantly lower than that mediated by MesobsFab.

A significant release of TNFα was observed during MesobsFab-mediated ADCC against MDA MB 231 monolayers at all E:T ratio but only at high E:T ratio on MDA-MB 231-derived spheroids. By contrast, no increase of TNFα release was observed during MesobsFab-mediated ADCC against HCC1806, except at high E:T ratio against HCC1806 monolayers (Figure 5B).

### *In vivo* Antitumor Efficacy of MesobsFab Against TNBC Tumors

**Figure 6:**
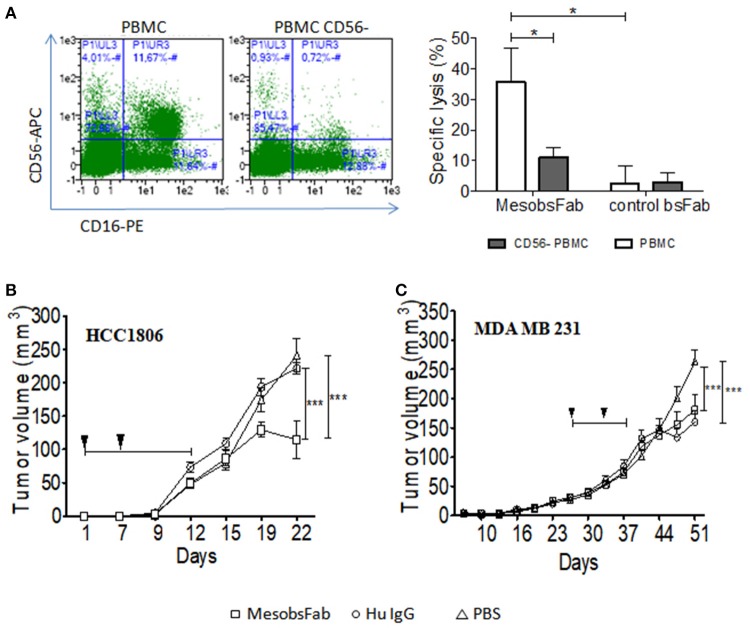
MesobsFab antitumoral efficacy *in vivo*. **(A)** Left panel: frequency of CD56+CD16+ cells in PBMC before and after depletion of CD56 cells. The percentage of CD56+CD16+ double-positive cells in the PBMC population is indicated. Cells were labeled with CD56-APC, CD16-PE antibodies. Right panel: flow cytometry-based ADCC assays were performed on 2D cell models using 40 nM of bsFab, unstimulated PBMC (white bar), or CD56-depleted PBMC (gray bar) as effector cells and HCC1806 as target cells (E:T ratio 25:1). CFSE-labeled target cells mortality was determined by staining with TO-PRO3. Specific cell lysis was determined using the formula:: [TEA CFSE+ TO-PRO3+ – TE CFSE+ TO-PRO3+/100 – ET CFSE+ TO-142 PRO+] with TEA = Target + Effector + Antibody, TE = Target+ Effector. Data are presented as mean ± SEM of four independent experiments (4 healthy donors). *P* values were determined by one-tailed unpaired *t*-test: ^*^P < 0.05; MesobsFab/PBMC vs. MesobsFab/PBMC CD56^−^; ^*^P < 0.05; MesobsFab vs. control bsFab. HCC1806 tumors **(B)** and MDA MB 231 tumor **(C)** growth in NSG mice that received unstimulated human PBMC twice (arrows) and treated with MesobsFab (5 mg/kg) every day for 12 days, irrelevant human IgG (5 mg/kg) 3 times a week or vehicle (PBS), (*n* = 5–6 mice/group). Duration of treatment is represented by the black line. Tumor growth was followed using a Vernier caliper. Data are presented as mean tumor volume ± SEM. *P* value were determined by two-way ANOVA with Bonferroni post tests. HCC1806: ^***^P < 0.001 MesobsFab *vs*. Hu IgG (D19 and D22); ^*^P < 0.05 MesobsFab *vs*. PBS (D19), ^***^P < 0.001 MesobsFab *vs*. Hu IgG (D22). MDA MB 231: ^*^P < 0.05 MesobsFab *vs*. PBS (D48), ^***^P < 0.001 MesobsFab vs. PBS (D51), ^***^P < 0.001 Hu IgG *vs*. PBS (D48 and D51).

As illustrated in Figure 6A, we first confirmed that MesobsFab retained its ADCC activity using unstimulated peripheral blood mononuclear cells (PBMC) as effector cells (E:T ratio = 25). As expected, depletion of NK cells led to a significant drop in activity, confirming the predominant role of this cells in MesobsFab cytotoxic activity. Next, we investigated the *in vivo* antitumor efficacy of MesobsFab in MDA MB 231 and HCC1806 orthotopic xenograft models in PBMC-humanized NSG mice. Based on preliminary experiments on tumor cell growth with or without PBMC, the antibody treatment and PBMC transplant protocol were adapted for each model. Untreated (PBS) or (human IgG + PBMC) treated mice were used as controls. During the treatment, MesobsFab was administered every day during 12 days to compensate for its short half-life compared to every 3-days for human IgG. As shown in Figure 6B, a significant and sustained retardation of HCC1806 tumor growth was observed in MesobsFab treated mice compared to control mice (*p* < 0.001). For MDA MB 231 mice models, a modest but significant (*p* < 0.001) slowdown in tumor growth was induced after treatment (from day 41) by MesobsFab compared to untreated mice but not to IgG-treated mice (Figure 6C). Monitoring of animal survival and weight curves indicated no apparent off target toxicity of MesobsFab, despite repeated injections but GvH effect started to appear in MBMC treated mice at day 22 and 48 for HCC1806 and MDA MB 231.

## Discussion

Despite a significant overall decrease in breast cancer-related mortality explained by major improvements in early detection and therapeutic strategies, triple negative breast cancers which are clinically defined by the absence of hormonal and HER2 receptors, remain a clinical challenge with limited therapeutic options and the worse overall survival. Thanks to continuous and major advance in immuno-oncology, new tracks of targeted therapies are being explored (26). Among these tracks, mesothelin has recently emerged as a “hot prospect” because of its restricted expression on mesothelial cells in healthy tissues and its over-expression in several aggressive cancers and notably in 30–70% of TNBC (6, 16, 27). Moreover, although controversial, the over-expression of MSLN in TNBC seems associated with a poor prognosis (18, 19). These features led to the development of different anti-MSLN therapeutic agents (16, 28). Amatuximab (MORAb-009) is currently the only unconjugated anti-MSLN monoclonal antibody examined in clinical trials but its therapeutic efficacy as single agent is disappointing (14), reinforcing the need to find new anti-MSLN antibody-based therapies.

In this context, we evaluated a bispecific antibody retargeting strategy aiming at engaging CD16 expressing effectors to tumor site in TNBC using a versatile Fab-like bispecific antibody format derived from llama single domain antibodies (21, 22). Endowed with original features such as a unique, specific, and high CD16 binding apparent affinity, this format has been shown to overcome limitations of monoclonal antibodies associated with low affinity for CD16 polymorphism sensitivity (21, 22). The previously characterized anti-MSLN single domain antibody A1 (23) was used to develop a Fab-like bispecific antibody, MesobsFab, which targets MSLN on tumor cells and CD16 expressing effector cells such as NK cells. Two TNBC cell lines, MDA MB 231 and HCC1806, with different epithelial/mesenchymal phenotypes and MSLN expression level were chosen as TNBC models.

We demonstrated that binding of membrane-bound MSLN by MesobsFab significantly decreased the invasiveness of both TNBC cells but had no effect on cell migration. Disruption of MSLN/MUC16 interaction by MesobsFab cannot explain this result because MUC16 expression has not been documented in breast cancer (29). A potential explanation of this result could be an indirect effect of MesobsFab on the MSLN-driven secretion of matrix metalloproteases, notably MMP-7 and MMP-9 which have been described as promoting tumor invasion (9, 11, 30, 31). Although some of our preliminary data indicate that invading HCC1806 cells secrete high amount of MMP-9 and that this secretion is significantly reduced by MesobsFab binding to MSLN, further investigation are required to delineate the impact of MesobsFab/MSLN interaction on MMPs secretion and invasive capacities in TNBC.

Compelling evidence has shown that spheroids, although lacking solid tumor complexity, recapitulate some of the morphological, histological, and physiological characteristics of solid tumors (32, 33). By mimicking the architecture of small avascularized tumor nodules, spheroids constitute attractive alternative models for testing the effectiveness of putative therapeutic molecules and for exploring some issues that cannot be addressed by conventional monolayer culture such as immune cell infiltration in tumors or the impact of the 3D organization of tumor cells on ADCC. In this study, multicellular tumor spheroids of TNBC were developed in parallel with conventional 2D cell monolayers. HCC1806- and MDA MB 231-derived spheroids displayed striking differences in terms of morphological and immunohistochemical characteristics as well as migration/invasion patterns and MSLN expression, reflecting the heterogeneity of TNBC. Given the critical impact of immune infiltration in these tumors we took benefit of our 3D culture models to investigate the capacity of MesobsFab to mediate NK cells recruitment and infiltration. Although spheroids are more and more used to mimic the impact of the tumor microenvironment on NK cell function, they are scarcely used to investigate the process of recruitment, infiltration and immunosurveillance. Infiltrated NK cells were detected by lightsheet fluorescence microscopy which allows live imaging and non-invasive optical sectioning of fluorescently labeled 3D samples (34, 35), thus bypassing the disadvantage associated with spheroids fixation and sectioning in immunohistochemistry analysis. A 3D analysis method based on Bayesian multiview deconvolution (36) was developed to quantify and localize any NK cells infiltrated into spheroids. We show that MesobsFab enhanced NK cell recruitment and increased the number and the probability of NK cell penetration in deeper distances compared to NK cell/spheroids alone. Unexpectedly, the control bsFab recruited as much NK cells as MesobsFab and favored NK cell infiltration. Similar results, but to a lesser extent, were observed with control human IgG which displays a lower affinity for CD16 compared to that of MesobsFab. Importantly, none of these control antibodies bind tumor cells nor trigger cytotoxic activity on HCC1806 spheroids. These counterintuitive results suggest that the monovalent engagement of CD16 on NK cells might contribute to the recruitment and infiltration of NK cells. Yet beyond the scope of this study, further investigations are currently ongoing to explain these results and better understand the dynamic of NK cell recruitment within the tumor environment.

We next assessed the ability of MesobsFab to trigger NK cell cytotoxicity against TNBC cells *in vitro*. For sake of comparison, we chose to keep similar experimental set up for 2D and 3D ADCC assays, using the same starting number of tumor cells, E:T and incubation time. We showed that MesobsFab elicited efficient and potent ADCC using unstimulated human NK cells against the two TNBC cells cultured in both 2D or 3D conditions. MesobsFab induced granzyme/perforin-mediated cytotoxicity and cytokine secretion in response to target cell recognition and CD16 engagement. However, the 3D organization of tumor cells proved to have an influence on the tumor cells susceptibility to NK cell cytotoxicity, on MesobsFab efficacy (maximal lysis) and potency (EC_50_) and on cytokine secretion in response to MesobsFab driven CD16 engagement. Thus, a higher non-specific lysis mediated by the control bsFab was observed on spheroids perhaps reflecting the recruitment and infiltration of NK observed in these conditions that might translate to increased natural cytotoxicity. The efficacy of MesobsFab was strongly dependent on the E:T ratio on both cell lines in 2D; this parameter appeared to have a lower impact in 3D, notably with MDA MB 231 cells. As well, a tremendous increase of IFNγ secretion was observed when tumor cells were grown as spheroids reflecting a strong NK cell activation mainly dependent on tumor cell/immune cell cross-linking. One potential explanation could be that the spatial environment and the high density of target cells in 3D format enhances NK cell activation by promoting MesobsFab mediated serial contacts between NK and tumor cells (37, 38) and/or the engagement of other activating receptors such as NKG2D or NKp46 which has been shown to trigger IFNγ secretion (39). Yet, MDA MB 231 cells express several NKG2D ligands (40). This finding is of interest as the presence of IFNγ in the tumor microenvironment promotes immune cells activation and tumor immunogenicity shaping (41). No increase in TNFα secretion was observed although Fauriat et al. (42) described an interrelationship between IFNγ and TNFα secretion. Importantly, the low level of cytokine secretion by NK cells induced by MesobsFab in the absence of target cells *in vitro* suggests that MesobsFab should not trigger any systemic activation of NK cells *in vivo* and should not induce cytokine storm. Moreover, we previously showed that the presence of neutrophils, expressing CD16B, a non-signaling GPI-linked version of the receptor, did not disturb NK cell-mediated ADCC, at least *in vitro* on tumor cell monolayers (22).

Validation of the *in vivo* efficacy of new immunotherapies remains a real brainteaser as these approaches require a functional immune system. In this respect, syngeneic transplantable models are attractive models but many differences between human and mouse immune systems represent a major limitation that might account for the regular differences experienced between pre-clinical and clinical immune-based studies. An extra level of complexity resides in the fact that overexpression of mesothelin in immunocompetent mice has been shown to inhibit tumor formation (43). For mimicking a human tumor/immune system context, the *in vivo* efficacy of MesobsFab was thus evaluated in a model of PBMC humanized mice generated by human PBMC engraftment and orthotopic MDA MB 231 or HCC1806 xenografts in severe immunodeficient mice (NSG). Despite the scarcity of human NK cells due to their short life span in such model, a significant slowdown of HCC1806 tumor growth mediated by Mesobsfab was observed. On the other hand, its impact on MDA MB 231 tumor growth did not reach significance relative to human IgG-treated mice. This result suggests that *in vivo* the anti- tumor efficacy of MesobsFab depends upon a threshold of MSLN density on target cells. This finding is at variance with those observed *in vitro* with tumor spheroids which suggested a low impact of MSLN expression level on MesobsFab efficacy. The efficacy of MesobsFab on MSLN-positive TNBC could be of particular interest as this subgroup was described to display a short interval to metastasis and high risk of developing brain metastasis (18). Further investigations on additional MSLN-positive TNBC cell lines will be necessary to confirm this result. Besides, although CD56 depletion of human PBMC confirmed the pivotal role of NK cells in the cytotoxic activity of MesobsFab, the potential role of other CD16-positive effector cells in the cytotoxic activity of MesobsFab could be an interesting issue to investigate as the importance of CD16 engagement has been recently reported for monocytes-mediated ADCC (44), neutrophil activation (45) or NK cell proliferation and memory-like cytotoxicity (46). As well, the use of more advanced models such as human IL-15 expressing NOG mice engrafted with human CD56+ cells derived from PBMC or human hematopoietic stem cells to sustain and amplify NK cells should be the next step to ascertain the *in vivo* efficacy of MesobsFab and investigate the long term effect of MesobsFab. As well, due to the scheduling of the treatment and the short *in vivo* half-life of MesobsFab (21), it can be assumed that it is no longer present during the last 15 days of the experiment. Strategies that significantly increase tumor retention can be envisaged such as continuous infusion, a process described for BiTEs (47) or the addition of an anti-human serum albumin sdAb to confer a long serum half-life to the bsFab format (48).

Collectively, our data support the potential of targeting mesothelin in mesothelin-positive TNBC using MesobsFab as a new targeted immunotherapy.

## Methods

### Cells

BT-474 (ATCC HTB-20), HCC1806 (ATCC® CRL-2335™), SK-BR-3 (ATCC® HTB-30™) and MDA-MB-231 (ATCC® HTB-26™) were purchased from ATCC. Ovarian cancer cells line, A1847 and human FcγRIIIA transfected Jurkat lymphoma T cells (Jurkat-huFcγRIIIA cells) were a gift of Dr N. Scholler, (SRI International, Ca USA) and Pr. Eric Vivier (Marseille, France), respectively, and were not authenticated. NK cells were isolated from human PBMCs as previously described (21). NK cells purity and activation status were determined by flow cytometry (MACSQuant® analyser (Miltenyi Biotec) after staining with PE-conjugated anti-CD3, anti-CD56, anti-CD16, anti-CD69, and anti-CD107 (Miltenyi Biotec). Mesothelin binding capacity of tumor cell lines was quantified by DAKO QIFIKIT (DAKO Cytomation), according to manufacturer's protocol using anti-human mesothelin monoclonal antibody K1 (GTX23362, GeneTex) as primary antibody. MSLN quantity was expressed as specific antibody-binding capacity units after substracting background from isotype control.

### Multicellular Tumor Spheroid Formation

Multicellular tumor spheroids were generated by seeding single cell suspension (5 × 10^3^ cells/well) with viability above 90% in Corning® Costar® 96 well round bottom ultra-low attachment plates and cultured in complete medium under standard conditions for 2–3 days. For longer incubation time, spheroids were fed every 4 days by carefully replacing half medium by fresh complete medium. Spheroids were stained at day 4, 8, and 14 with propidium iodide (1 μg/mL, Miltenyi Biotec) for 3 h at 37°C and with Hoechst 33342 (ThermoFisher) at 1 mg/mL for 30 min at 37°C for detection of necrotic core and proliferation zones, respectively. Spheroids were imaged using an inverted transmitted light/fluorescence microscope (EVOS FL Auto Imaging system, Life Technologies). Spheroid diameters were determined using ImageJ software (NIH, Bethesda, MD; http://imagej.nih.gov/ij and their size was calculated using the formula: V = 4/3πr^3^. Spheroid size reproducibility was determined using the percent of coefficient of variation [CV (%)]. A CV inferior to 15% indicated a homogenous sample.

### Immunohistochemistry on Spheroids

Spheroids were fixed for 1 h at room temperature in 4% methanol free-formaldehyde, embedded in 5% agarose before embedding in paraffin for immunostaining analysis. An antigen retrieval step at acidic pH (pH 6) was performed before immunohistochemical staining of spheroid sections (4 μm) for all antibodies except for anti E-cadherin antibody (pH 9). Staining was performed with anti-Ki-67 (1:50, Sigma-Aldrich), anti-E-cadherin (1:250, Abcam) and anti-vimentine (1:400, Abcam) and immunoreactivities were detected using the VECTASTAIN® ABC Kit (Vector Laboratories) according to the manufacturer's protocol and liquid diaminobenzidine substrate chromogen system (Dako; K3468). Counter-staining in Mayer Hematoxilin followed by a bluing step in 0.1% sodium bicarbonate buffer was performed before mounting of the sections.

### 3D Migration and Invasion Assays

For the migration assays, 4 day-spheroids were transferred into a 96 well flat bottom plate (Clear flat bottom surface Corning® Costar®, Life Sciences) pre-coated with 1% gelatin (Sigma-Aldrich) in DMEM-5% FBS (MDA-MB 231) or RPMI-1% FBS (HCC1806) medium. For the invasion assays, 4-day spheroids were embedded in 50% Matrigel (BD Matrigel™ Basement Membrane Matrix Phenol Red free, BD Biosciences). Once Matrigel solidified, 100 μl of complete culture medium was added on top. Images of the spheroids were captured during 72 h and analyzed using ImageJ software.

### 2D Migration and Invasion Assays

Two-dimensional migration was investigated using 8 μm FluoroBlok™ (Corning® Fluoroblok™ Cell Culture Inserts, BD biosciences). Two days before the assay, tumor cells were stained with 10 μM carboxyfluorescein diacetate succidimyl ester (CellTrace™ CFSE Cell Proliferation kit, ThermoFisher Scientific) for 20 min at 37°C. One day before, cell cycle synchronization was performed by serum starvation. The upper chambers were seeded with CFSE-labeled tumor cells (10^5^) in RPMI medium with or without bsFab (50 nM). Chemotactic gradient was created by addition of 5% FBS medium in the lower chamber. The fluorescence in the bottom chamber measured at 6 and 24 h on a fluorescent plate reader (Tecan Infinite® M1000—Life Technologies) at 521 nm corresponds to the migrating cells.

Invasion assays were performed as above using 8 μm FluoroBlock™ inserts pre-coated with 1 mg/ml Matrigel.

For each experiment, fluorescence values for the control + 5% FBS were set as 100% migration/invasion. The ratiometric results were expressed relative to this sample.

### *In vitro* NK Cell-Mediated ADCC Assays

Two-dimensional *in vitro* assays with fresh human NK cells were performed using CellTiter-Glo Luminescent Cell Viability Assay (Promega) as previously described (22). 3D ADCC assays were performed by flow cytometry using CFSE (1 μM)-prestained MDA-MB 231 or HCC1806 spheroids. The size homogeneity of spheroids was verified by imaging prior cytotoxicity assays. At day 2, antibodies (5 nM) and purified human NK cells at various E/T ratios were added in the medium. After overnight incubation, washed spheroids were dissociated with TrypLE express (LifeTechnologies) for 5–10 min at 37°C. Cells were immunostained with an APC-conjugated anti-CD45 (1/40, Miltenyi Biotec), then with 1 μM SYTOX® blue (Molecular Probes, LifeTechnologies) for 5–20 min, in the dark. Quadrant statistics was applied on the dot plots. CD45-APC^negative^ tumor cells were gated and remaining viable tumor cells were identified as APC^−^/CFSE^+^/SYTOX Blue^−^. Percent specific cytotoxicity was calculated as follows: 1 – [TEA ^CFSE+SYTOX−^ – TE ^CFSE+SYTOX−^/T ^CFSE+SYTOX−^] with TEA = Target + Effector + Antibody, TE = Target + Effector, and T = Target.

### *In vitro* PBMC-Mediated ADCC Assays

Human PBMCs were isolated from healthy donors via Ficoll Histopaque® (Sigma-Aldrich) density gradient centrifugation. The NK cell fraction was depleted by incubation of PBMC with MACS® CD56 MicroBeads, human (Miltenyi, #130-050-401) followed by manual separation using MACS LD Columns (Miltenyi) following the manufacturer's instructions. The CD56*-depleted* fraction was collected and stained with anti-human CD3-PE, anti-human CD16-PE, and anti-human CD56-APC antibodies (Miltenyi) for 30 min at 4°C.

Flow cytometry-based cytotoxicity assays were performed with PBMC and CD56-depleted PBMC. Target cells (HCC1806, SK-BR-3) were stained with 1 μM carboxyfluorescein diacetate succinimidyl ester (CFSE—LifeTechnologies) according to manufacturer's instructions. Following labeling, the cells were washed and analyzed by flow cytometry for viability (>95%). Effector cells were preincubated with antibodies (40 nM) for 20 min at 37°C then co-incubated with CFSE-labeled tumor cells (4 × 10^4^/well) at effector/target (E:T) ratio of 25 in RPMI-GlutaMAX™ medium supplemented with 10% FBS for 3 h. Cells were washed with PBS/BSA 2%, stained with TO-PRO®-3 (1/1000) (LifeTechnologies) and analyzed by flow cytometry on a MACSQuant cytometer. Gating on TO-PRO®-3/CFSE double positive cells indicated dead target cells in percentage (%). Percent specific cytotoxicity was calculated as follows: [TEA ^CFSE+TO−PRO3+^ – TE ^CFSE+TO−PRO3+^/100 – TE ^CFSE+TO−PRO3+^] with TEA = Target + Effector + Antibody, TE = Target + Effector.

### NK Cell Activation Assays

Supernatants of 2D and 3D ADCC assays were harvested and frozen at −20°C. Secreted human IFNγ, TNFα, and granzyme B were measured by ELISA using the READY-SET-GO human IFNγ or TNFα kits or the Human Granzyme B platinum ELISA kit as described by the manufacturer (eBioscience).

### 3D NK Cells Recruitment

Purified human NK cells (2 × 10^6^ cells) were labeled with 0.4% PKH26 (MINI26, Sigma Aldrich) during 5 min at 4°C, in the dark and with stirring. At day 2, CFSE stained HCC1806 spheroids were mixed with PKH26-labeled NK cells (12 500 cells) (E:T = 2.5:1) and antibodies (50 nM). After 48 h incubation, imaging was performed using a LightSheet Z.1 microscope (Carl Zeiss Microscopy, GmbH, Jena, Germany) along 4 different angular views, every 90° for 3D reconstruction using the Multiview Reconstruction plugin for Fiji (36) and automated using a jython script. View alignments were performed using the RANSAC algorithm and fluorescent beads as interest-point. The fusions were performed with a mean of the different views. Jython scripts for Fiji were used for measuring NK cell infiltration. For each spheroid, a mask was created using an intensity threshold on the CFSE-labeled tumor image and manuals corrections to remove holes and protuberance to respect the spheroid shape. PKH26-labeled NK cells were detected with the Regional Min and Max 3D (Morpholibj plugin) (49) after a Laplacian of Gaussian filtering (with a size 6 μm, the FeatureJ plugin (https://imagescience.org/meijering/software/featurej/). The local maxima detections were filtered by removing the detection outside the spheroid mask and by an intensity threshold of 1 (on the Laplacian of Gaussian image). The NK cells depths were measured by calculating the Chamfer Distance Map 3D of the spheroid mask with the MorpholibJ plugin (26).

### *In vivo* Tumor Growth Studies

All animal experiments were performed in agreement with the French Guidelines for animal handling and approved by the local ethic committee (C2EA14) for Animal Experimentation (Agreement no. APAFIS#13350-2018012313504256v4). HCC1806 (2 × 10^5^ cells/mice) and MDA MB 231 (1 × 10^6^ cells/mice) in a 1/2 (v/v) Matrigel (Corning Life Sciences, Bedford, MA, USA) suspension were injected into mammary fat pads of 8-week-old female NSG mice. For HCC1806 models, freshly purified human PBMCs (15 × 10^6^ cells/mice) pre-incubated with MesobsFab (5 mg/kg, *n* = 6 mice/group) or control human IgG (5 mg/kg, *n* = 6 mice/group) were injected intravenously (i.v) at days 1 and 7 post-graft. From day 2 to 12, MesobsFab and hu IgG (5 mg/kg) were injected (i.p) daily and every 3 days, respectively. For MDA MB 231 models, mice were randomly divided into treatment groups (*n* = 6 mice/group) when tumors reached an average of 30–40 mm^3^ (28 days post-graft) and human PBMCs pre-incubated with Mesobsfab or human IgG (5 mg/kg) were injected i.v. During the next 10 days, MesobsFab and hu IgG (5 mg/kg) were injected i.p daily and every 3 days, respectively. A second injection i.v. of PBMCs pre-incubated with antibodies was performed 7 days after the first one. Tumor growth was monitored by digital caliper measurements and by calculating volumes (length × width^2^ × π/6).

### Statistical Analysis

In all graphs data are presented as mean ± SEM. One-tailed or two-tailed unpaired Student's *t*-test (*in vitro* experiments) and Two-Way ANOVA with Bonferroni post tests (*in vivo*) were performed using a 95% confidence interval using Prism 5 (GraphPad software). *P*-values < 0.05 were considered significant.

## Ethics Statement

This study was carried out in accordance with the French guidelines for animal handling and protection. The protocol was approved by the animal ethics committee C2EA14 (Agreement no. APAFIS#13350-2018012313504256-v4).

## Author Contributions

JD and BK: conceptualization. JD, RF-F, and LG: methodology. JD and LG: investigation. EJ, AG, and RC: animal studies. PC and BK: supervision. JD, PC, and BK: writing. DB and BK: funding acquisition.

### Conflict of Interest Statement

The authors declare that the research was conducted in the absence of any commercial or financial relationships that could be construed as a potential conflict of interest.
