# Supplementary material for: A Bispecific Antibody-Based Approach for Targeting Mesothelin in Triple Negative Breast Cancer
Source: Front Immunol. 2019 Jul 10;10:1593. doi: 10.3389/fimmu.2019.01593 (PMC6636429; doi:10.3389/fimmu.2019.01593)
Supplement: Supplementary file 2 [file Data_Sheet_1.docx]

**Supplementary material**

**Supplementary Methods**

**Cells**

All cell lines were from the American Type Culture Collection (ATCC), except A1847 (generous gift of N. Scholler, SRI International, USA) and Jurkat-huFcγRIIIa/γ cells (generous gift of E. Vivier (CIML, Marseille, France)). All cells were maintained in a humidified environment at 37°C and 5% CO_2_ during culture, routinely tested for mycoplasmas (MycoAlert Mycoplasma Detection Kit, Lonza) and cultured for no more than 3 months after thawing. Human peripheral blood mononuclear cells (PBMCs) were isolated from platelet-leukocyte concentrated fraction of whole blood from healthy donors (Etablissement Français du Sang (EFS), Marseille, France) by Ficoll LSM 1077 (PAA) gradient centrifugation.

**bsFab production and biochemical characterization**

Anti-meso sdAb, anti-CD16.21 and anti-gp120 sdAb were previously selected by phage display after immunization of lamas and characterized for their binding properties. MesobsFab and control bsFab were constructed by cloning the cDNA of the anti-Meso sdAb (Meso.A1) [[1](#_ENREF_1)] or anti-gp120 sdAb (clone JM4) [[2](#_ENREF_2)] into the bicistronic expression vector pBAT14 upstream the cDNA of the human anti-FcγRIII sdAb (CD16.21) cDNA [[3](#_ENREF_3)]. The resulting bispecific plasmids allow the expression of bsFab containing either the anti-Meso sdAb or the anti-gp120 sdAb fused to the human Ck domain and the CD16.21 sdAb fused to the human CH1 domain. Both bsFab were produced in *E.coli* (*DH5α* strain) periplasm and purified by affinity chromatography on IgG-CH1 matrix, followed by affinity chromatography on LC-kappa (Hu) matrix (Capture Select®, BAC BV) as previously described [[4](#_ENREF_4)] . Purity of bsFab was evaluated by SDS-PAGE on Mini-PROTEAN^®^ TGX Stain-Free precast gel under non reducing conditions and immunoblotting. Detection on Western blot was performed using an anti-HIS-HRP (1/5000, Miltenyi) or anti-Flag-HRP (1/5000). Apparent affinities were determined by flow cytometry as previously described [[4](#_ENREF_4),[5](#_ENREF_5)] using 2x10^5^ Jurkat-huFcγRIIIA, HCC1806 and A1847 cells and a range of biotinylated antibodies concentration (0.05 nM to 2000 nM). Binding was detected using PE-labeled streptavidin. The binding of MesobsFab, mAb K1 and control bsFab (100 nM) was evaluated by flow cytometry on mesothelin positive cells (HCC1806) and mesothelin-negative cells such as BT474. Binding was detected using PE-labeled streptavidin. Immunofluorescent competition assays were performed by incubating 2x10^5^ HCC1806 cells with serial dilutions of either MesobsFab, mAb K1 or control bsFab (competitor) and biotinylated MesobsFab (tracer, 20 nM). The binding of biot-MesobsFab was detected with PE-labeled streptavidin.

**NK cell recruitment**

Five to six MCTS of homogenous volume and shape per condition were embedded in a droplet of warm (37-40°C) 1% low-gelling agarose containing fluorescent beads (1:1000, Fluorescent Estapor® microspheres 0.52 µm, Ex/Em : 505/515nm) and pipetted into thin glass capillaries (0.8 mm). Data acquisition was performed using a 488 nm laser for CFSE-labeled tumor cells and fluorescent beads or a 561 nm laser for PKH-26 labeled NK cells with a 20x/1.0 W Plan-AOCHROMAT objective. For analysis, the images were down sampled 4x in the x and y axis with the z matching the x and y resolutions.

**Supplementary Figures**

**Figure S1 - Biochemical characterization of MesobsFab**

A) Schematic representation of bsFab. Dark grey, Cκ and CH1 constant domains of human IgG1; Light grey, anti-CD16 sdAb (clone CD16.21); White, anti-human MSLN sdAb (clone A1) or anti-human control sdAb (gp120); C, H and F, c-myc, hexahistidine and Flag Tag, respectively. B) SDS-PAGE (left) and Western Blot (right) analysis of purified MesobsFab : lane 1, MesobsFab 1µg; lane 2, MesobsFab 0.5µg; lane 3, Precision Plus Protein Unstained Protein Standard (BioRad); lane 4, SeeBlue Plus2 Pre-stained Protein Standard (LifeTechnology). C) Binding curves of serial dilutions of MesobsFab on Jurkat-CD16 cells (left) and HCC1806 (right). HER2bsFab [[5](#_ENREF_5)] and anti-Meso mAb K1 have been added as controls for CD16 or mesothelin binding respectively. D) Binding of MesobsFab and mAb K1 (100 nM) on Mesothelin-positive lines. E) Binding of MesobsFab, mAb K1 and control bsFab (100 nM) on mesotheline-negative (BT474) and mesothelin-positive (HCC1806) cell lines. F) Inhibition of biotinylated MesobsFab binding to HCC1806 by serial dilutions of control bsFab, MesobsFab and mAb K1. Binding was detected using PE-labeled streptavidin.


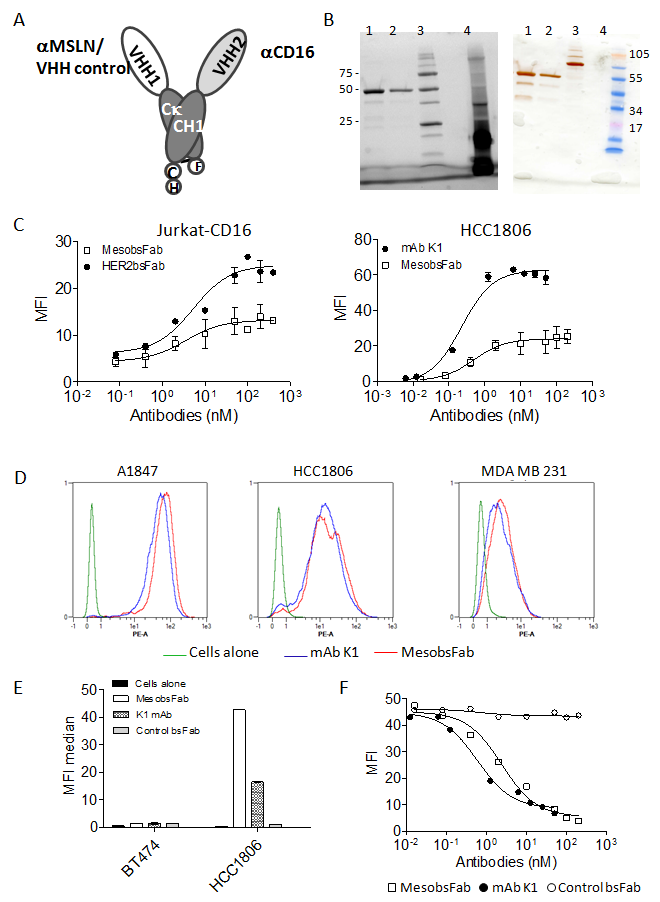


**Figure S2 - Characteristics of TNBC spheroids**

A) Qualitative analysis of proliferation and necrotic zones into spheroid. Proliferation and necrotic zones are determined using Hoechst (blue) and propidium iodure staining (red), respectively. B-C) Comparison of necrotic zone evolution on day 4 (D4) and day 8 (D8) spheroids. Images are acquired using an EVOS FL Auto microscope. Scale : 500 µm.


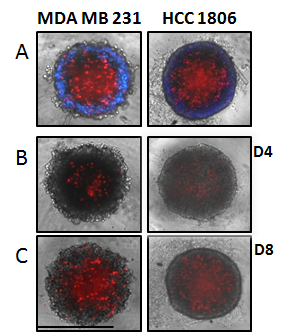


**Figure S3 - Granzyme B secretion in 2D and 3D ADCC assays and cytotoxic specificity of MesobsFab**

A) Granzyme B secretion was quantified by ELISA in the medium harvested from 2D and 3D ADCC assays (Fig. 4B) (E/T: 10/1). Assays were performed in triplicate and are representative of at least three independent donors. Data represent the mean ± SEM. Data were analyzed by two-tailed Student's t-test. MDA MB 231: ** *P* < 0.01 *** *P* < 0.001

B) ADCC assays were performed on 2D and 3D cell models using NK cells as effector cells and against mesothelin-negative cell line, BT474 as target cells (E/T ratio 10:1) in the presence of irrelevant bsFab or MesobsFab 5 nM (n=3). Target cell viability was determined by CellTiter-Glo viability assay for 2D ADCC or by flow cytometry after spheroid dissociation for 3D ADCC. Data represent the mean +/- SEM.


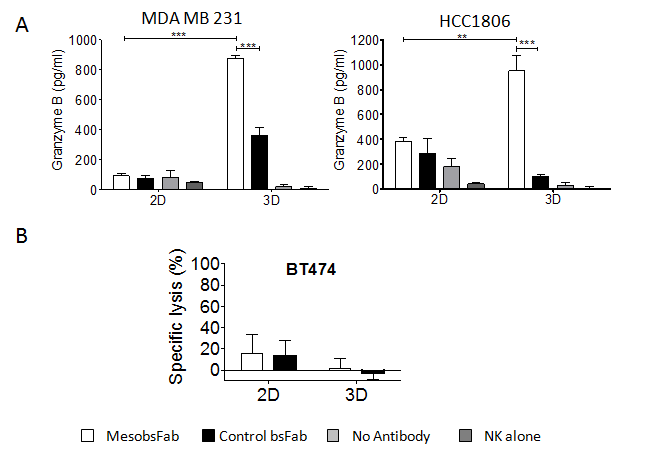


**Table S1 - K_dapp_ values of MesobsFab and monoclonal anti-mesothelin antibody, K1.**

**Movie S1 - 3D analysis of NK cell recruitment**

Movie, made with Imaris ver. 9.1.2, illustrates the different steps of the 3D analysis of NK cell recruitment shown in figure 3A. The different views represent (i) a 3D rotation of HCC1806 spheroid (ii) z-stack of the PKH-26-labeled NK cells, (iii) z-stack of these cells after a Laplacien of Gaussian filter, (iv) the detected NK cells in red superimposed with the initial z-stack, (v) z-stack of the CFSE-labeled tumor cells, (vi) z-stack of the tumor cells mask with a color code for mask distance map (from 0 (surface-blue) to 160 (inside-pink) µm). Then the movie shows the detected NK cells with a color code corresponding to the cells depth.

1. Prantner AM, Turini M, Kerfelec B, Joshi S, Baty D, et al. (2015) Anti-Mesothelin Nanobodies for Both Conventional and Nanoparticle-Based Biomedical Applications. J Biomed Nanotechnol 11: 1201-1212.

2. Matz J, Kessler P, Bouchet J, Combes O, Ramos OH, et al. (2013) Straightforward selection of broadly neutralizing single-domain antibodies targeting the conserved CD4 and coreceptor binding sites of HIV-1 gp120. J Virol 87: 1137-1149.

3. Behar G, Siberil S, Groulet A, Chames P, Pugniere M, et al. (2008) Isolation and characterization of anti-FcgammaRIII (CD16) llama single-domain antibodies that activate natural killer cells. Protein Eng Des Sel 21: 1-10.

4. Rozan C, Cornillon A, Petiard C, Chartier M, Behar G, et al. (2013) Single-domain antibody-based and linker-free bispecific antibodies targeting FcgammaRIII induce potent antitumor activity without recruiting regulatory T cells. Mol Cancer Ther 12: 1481-1491.

5. Turini M, Chames P, Bruhns P, Baty D, Kerfelec B (2014) A FcgammaRIII-engaging bispecific antibody expands the range of HER2-expressing breast tumors eligible to antibody therapy. Oncotarget.
